# Internal environment of footwear is a risk factor for tinea pedis

**DOI:** 10.1111/1346-8138.15060

**Published:** 2019-08-22

**Authors:** Yukio Sasagawa

**Affiliations:** ^1^ Sasagawa Dermatological Clinic Osaka Japan

**Keywords:** footwear, humidity, shoes, temperature, tinea pedis

## Abstract

The relation between tinea pedis and the internal environment of footwear has not been scientifically proven. This study aimed to determine whether the internal environment of footwear affects the incidence of tinea pedis and tinea unguium. This cross‐sectional, observatory study involved 420 outpatients who were categorized into non‐tinea, tinea pedis or tinea unguium groups based on mycological analysis. External climatic conditions, and temperature, humidity and dew points inside the patients’ footwear were recorded. Univariate and multivariate analyses were used to determine independent risk factors for tinea pedis and tinea unguium. A significant correlation was found between high temperature/high humidity and dew point of the internal environment of the footwear and the season. Furthermore, those who wore footwear with internal environments characterized by high temperature, high humidity, high‐temperature/high‐humidity and high dew point values had a significantly higher incidence of tinea pedis. The internal dew point correlated with the incidence of tinea pedis, whereas the external temperature correlated with the incidence of tinea unguium. The internal humidity and dew point of footwear as well as the frequency with which footwear with a high‐temperature/high‐humidity internal environment were worn was significantly higher in men than in women. In conclusion, the internal environment of footwear is a risk factor for tinea pedis, and this environment is affected by the season. Moreover, the frequency of tinea pedis among men is related to the internal environment of footwear. The dew point is an appropriate index for evaluating temperature and humidity in relation to tinea pedis.

## Introduction

For many years, epidemiological studies and clinical experience have indicated that a relationship exists between footwear and tinea pedis (TP).^1^, ^2^ The onset of TP is also likely to be more affected by the internal environment of the footwear enveloping the feet than the climate outside the footwear. However, this relationship has not been directly proven in any study, and data on this relationship are lacking.

Several risk factors have been reported for TP and tinea unguium (TU).^1^, ^2^, ^3^, ^4^, ^5^, ^6^ The risk factors include age, male sex, climate (high temperature and humidity), footwear, exercise, use of public facilities, home infections and incidence of certain diseases, such as obesity, diabetes, vascular disorders, and bone and joint diseases.^3^, ^4^, ^5^, ^6^, ^7^, ^8^, ^9^, ^10^ Furthermore, the proliferation of dermatophytes also varies with the season.^11^ Although temperature and humidity are considered risk factors for TP,^11^ studies investigating their association with dew point, an index of the degree of moisture contained in air, which is not affected by temperature, have not been conducted. The dew point is a good indicator of high temperature and humidity in the environment and can be used to demonstrate the effect of these environmental factors on dermatophytes. Although high temperature and humidity are risk factors for TP, these are currently not well defined.

The internal environment of footwear is determined by physical factors of the footwear (temperature, humidity, ventilation, heat conduction, footwear design, structure and materials), human physical factors (underlying diseases, physiological characteristics and human body structure), socks and environmental factors outside the footwear (local climate and ground environment).^12^ Therefore, this study aimed to determine whether there is a relationship between footwear and TP. Moreover, this study sought to determine whether the internal environment of the footwear is influenced by seasons and whether any of the known risk factors, such as age and sex, are also related to the internal environment of the footwear.

## Methods

### Subjects

In this observational study, 420 outpatients who wore footwear and who presented for examination at the Sasagawa Dermatological Clinic (Osaka, Japan) between 16 May 2011 and 28 November 2014 were enrolled.

Based on clinical assessment, subjects with fungal foot disease were classified into TP only, TU only and both (TP only and TU only) (three‐group classification). Subjects were also classified into TP (TP only and both) and TU (TU only and both) in a two‐group classification. For this study, fungal foot disease was considered as including TP only, TU only, or both TP and TU. In addition, the study cohort was divided into three age groups: group I, less than 30 years; group II, 30 years or more and less than 60 years; and group III, 60 years or more. Patient footwear was categorized as follows: (i) natural leather; (ii) synthetic leather; (iii) sneakers; (iv) cloth footwear; (v) sandals; (vi) slippers; and (vii) boots.

### Clinical assessment

Clinical assessments included follow‐up examinations, completion of a medical questionnaire and mycological analysis of samples collected from the infected sites. The toenails and feet were examined, and information about the symptoms or characteristics of TU or TP were recorded. Demographic data such as sex, age and employment were also recorded.

### Specimen collection and mycological testing

Skin and toenail scraping smears were collected from patients diagnosed with TP and TU and submitted to mycological testing. Samples were placed on slides and examined with direct microscopy after addition of 15% potassium hydroxide in 40% dimethylsulfoxide solution, using an optical binocular microscope (Olympus model 217577; Olympus, Tokyo, Japan).

### Temperature, humidity and dew point at measurement sites on the feet

Temperature, humidity and dew point inside the footwear of all patients were measured using an automatic temperature and humidity data logger monitor (DS1923, iButton loggers; Maxim Integrated, San Jose, CA, USA). The loggers were positioned on the dorsal side of the feet between the third and fourth toes. Humidity in the fourth interdigital space was also measured in 10 male subjects.

Data were recorded while subjects were wearing their footwear, once each minute for a total of 15 min. All measurements were taken while the patients were wearing socks to avoid direct contact with the skin. Patients remained at rest in a seated position for 15 min before measurements were made. Subjects remained at rest in a seated position for at least an additional 15 min after commencement of measurements. Dew points were calculated automatically using software in the logger and were based on temperature and humidity values. A high‐temperature/high‐humidity environment inside the footwear was defined as a temperature of 32°C or more and humidity of 80% or more.

### Climatic factors

External temperature and relative humidity measurements were obtained from climate data records for the Osaka region compiled by the Ministry of Land, Infrastructure, Transport and Tourism, and the Japan Meteorological Agency. In accordance with the designations used by the Japan Meteorological Agency, spring was defined as March to May, summer as June to August, fall as September to November, and winter as December to February. Fall and summer were collectively referred to as the warm season and winter and spring as the cold season.

### Statistical analysis

All statistical analyses were performed using IBM SPSS Statistics 22.0 software (Windows version; IBM, Armonk, NY, USA). Data are presented as mean ± standard deviation. Different tests were used for each analysis. The optimum cut‐off point was determined by the receiver–operator curve (ROC) analysis that separates TP and non‐TP. Details of the tests used are described in Methods S1. The significance level of the statistical tests was set to 0.05.

### Ethical considerations

This study was conducted in accordance with the ethical guidelines for epidemiological studies designated by the Ministry of Health, Labor and Welfare, and the Ministry of Education, Culture, Sports, Science and Technology after receiving approval from the Institutional Review Board of The Japanese Association for the Promotion of State‐of‐the‐Art in Medicine. All patients (or legal guardians for minors) provided written informed consent.

## Results

### Demographic and clinical characteristics

Overall, 420 participants (190 men, 45.23%; 230 women, 54.76%) were enrolled in this study. In terms of age demographics, 44.5% of subjects were in the working age group (Table S1). Among the 420 participants, 207 (49.2%) had fungal foot disease. The specific types of fungal foot disease were as follows: 115 patients (55.6%) had TP only, 42 patients (20.3%) had TU only, and 50 patients (24.2%) had both (TP only and TU only) (three‐group classification); 165 cases had TP (TP only and both) and 92 cases had TU (TU only and both) (two‐group classification).

The incidence of TP was significantly higher in men than in women (53.9% men, 46.1% women; *P* < 0.01). The percentage of men with TP only was significantly higher than that of women with TP only (*P* < 0.05). The prevalence of fungal foot disease was significantly higher in men than in women (*P* < 0.05). There was no sex‐based difference in the incidence of TU. The subjects were mainly office workers in the working age group, and the incidence of TP was 39.1%. For TU, there was no difference in the incidence between the two sexes.

### Climate factors and internal footwear environment

A significant correlation was found between the internal environment of the footwear and the external environment (mean temperature, *r* = 0.695; mean humidity, *r* = 0.522; *P* < 0.001). Furthermore, the mean temperature inside the footwear changed with the season, which was 29.9°C ± 3.4°C in spring, 32.6°C ± 1.5°C in summer, 31.5°C ± 2.6°C in fall and 26.5°C ± 4.1°C in winter. When the four seasons were grouped into two temperature extremes, the mean temperature inside the footwear was significantly higher (*P* < 0.001) in fall and summer (warm season, 32.0°C ± 2.3°C) than in spring and winter (cold season, 28.3°C ± 4.1°C). The humidity inside the footwear was also significantly higher (*P* < 0.001) in the warm season (76.7% ± 9.8%) than in the cold season (69.6% ± 10.2%). A similar trend was found for the dew point (warm season, 27.2°C ± 3.8°C; cold season, 21.9°C ± 5.5°C; *P* < 0.001) (Fig. S1). The number of footwear with a high‐temperature/high‐humidity internal environment was significantly greater during the warm season (*n* = 92/281, 32.7%) than during the cold season (*n* = 8/113, 7.1%; *P* < 0.001). These results show that the local climate affects the internal environment of the footwear.

### Footwear type and internal footwear environment

There were significant differences in temperature, humidity and dew point values among the seven types of footwear. Cloth footwear had the highest temperature, humidity and dew point values (32.2°C ± 1.4°C, 82.3% ± 8.0% and 28.7°C ± 2.0°C, respectively) and was therefore prone to mustiness (Fig. S2). Sandals and slippers had the best ventilation, with low temperature, humidity and dew point values.

The temperature, humidity and dew point values of closed footwear (31.8°C ± 2.6°C, 77.0% ± 9.4% and 27.1°C ± 3.8°C, respectively) were significantly higher than those of open footwear (29.7°C ± 3.5°C, 68.4% ± 9.2% and 23.1°C ± 5.3°C, respectively; *P* < 0.01; Table 1).

**Table 1:** Internal environment of different footwear types

	Temperature (°C)	Humidity (%)	Dew point (°C)
Natural leather (*n* = 51)	31.1 ± 3.1	76.2 ± 10.6	26.2 ± 4.7
Artificial leather (*n* = 129)	31.1 ± 3.4	75.9 ± 10.3	26.2 ± 5.1
Sneakers (*n* = 131)	31.6 ± 2.9	75.6 ± 9.5	26.6 ± 4.2
Cloth shoes (*n* = 10)	32.2 ± 1.4	82.3 ± 8.0	28.7 ± 2.0
Sandals (*n* = 18)	30.3 ± 2.4	72.6 ± 9.3	24.7 ± 3.9
Slippers (*n* = 6)	29.4 ± 4.2	68.4 ± 8.1	22.8 ± 5.8
Boots (*n* = 49)	28.4 ± 4.3	69.0 ± 12.6	21.8 ± 6.0
Closed (leather, artificial leather, *n* = 119)	31.8 ± 2.6	77.0 ± 9.4	27.1 ± 3.8
Open (sandals, slippers; *n* = 13)	29.7 ± 3.5	68.4 ± 9.2	23.1 ± 5.3

The seven types of footwear (natural leather shoes, synthetic leather shoes, sneakers, cloth shoes, sandals, slippers and boots) showed significant differences in temperature, humidity and dew point (*P* < 0.001) values. Cloth shoes had the highest temperature, humidity and dew point values, whereas sandals and slippers had lower temperature, humidity and dew point values. Closed footwear, such as that made of natural and synthetic leather, are commonly worn by business people and had significantly higher temperature, humidity and dew point values than the open sandals and slippers (*P* < 0.01).

Overall, the footwear with a high‐temperature/high‐humidity environment were more commonly worn by men (29.5%) than by women (20.4%, *P* < 0.05). The dew point was also higher among the footwear worn by men (26.5°C ± 4.3°C) than by women (25.0°C ± 5.3°C, *P* < 0.05). The types of footwear most often worn by office workers were synthetic leather (32.7%) and natural leather (12.9%). The types of footwear often worn by men were sneakers (16.8%), synthetic leather (14.2%) and natural leather (10.0%), whereas those worn by women were synthetic leather (20.3%), sneakers (13.5%) and natural leather (9%).

### Sex and internal footwear environment

Average humidity in footwear is 73.84% ± 10.19% (women, 71.96% ± 10.41%; men, 77.25% ± 8.90%). The humidity was significantly higher in men than in women (*P* < 0.05).

### Risk factors for TP and TU

**Figure 1:**
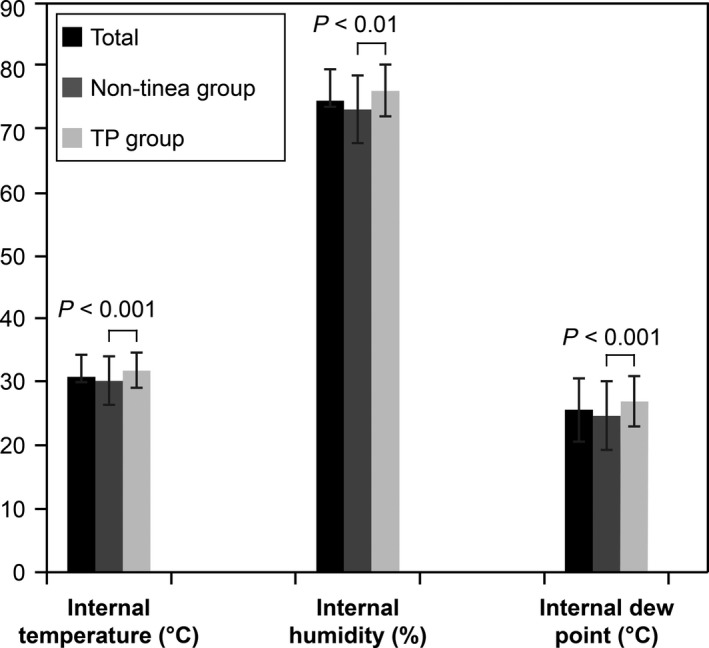
Internal temperature, internal humidity and internal dew point values of the footwear. Welch's two‐tailed *t*‐test was used to assess the internal temperature, internal humidity and internal dew point values, expressed as mean ± standard deviation. The internal environment of the footwear in those with tinea pedis (TP) had a significantly higher temperature (31.8°C ± 2.7°C, *P* < 0.001), humidity (76.1% ± 9.3%, *P* = 0.007) and dew point (26.9°C ± 4.0°C, *P* < 0.001) values.

The footwear of those with TP registered significantly higher temperature (31.8°C ± 2.7°C; *P* < 0.001), humidity (76.1% ± 9.3%, *P* < 0.001) and dew point (26.9°C ± 4.0°C, *P* < 0.001; Fig. 1) values compared with those of the non‐tinea. Furthermore, the percentage of footwear with high‐temperature/high‐humidity environment was also highest in the TP (30.9% vs 18.8% in the non‐tinea; *P* < 0.01).

The results of the univariate analysis for TP indicated that all variables were significant (Table 2), except for the mean monthly humidity in the local area. The footwear of those with TU registered significantly higher values for external temperature (odds ratio [OR], 1.10; 95% confidence interval [CI], 1.05–1.14; *P* < 0.001) and internal dew point (OR, 1.07; 95% CI, 1.01–1.13; *P* < 0.05; Table S2). The fungal foot disease also showed significantly higher values for temperature (OR, 1.15; 95% CI, 1.08–1.23; *P* < 0.001), humidity (OR, 1.03; 95% CI, 1.01–1.05; *P* < 0.01), dew point (OR, 1.10; 95% CI, 1.05–1.14; *P* < 0.001) and high‐temperature/high‐humidity (OR, 1.89; 95% CI, 1.20–2.98; *P* < 0.01; Table S3).

**Table 2:** Tinea pedis risk factor analysis

Variable	Univariate analysis			Multivariate analysis		
	Odds ratio	95% CI	*P*	Odds ratio	95% CI	*P*
Sex (men)	1.80	1.19–2.71	<0.01	–	–	<0.01
Age groups						
Total	–	–	<0.001	1.00 (reference)	1.00 (reference)	–
<30 years	1.00 (reference)	1.00 (reference)	–	9.16	1.11–75.85	<0.05
≥30 and <60 years	2.18	1.21–3.91	<0.01	20.26	2.48–165.76	<0.01
≥60 years	5.73	3.06–10.73	<0.001			
Internal high temperature/high humidity	1.93	1.20–3.12	<0.01			
Internal dew point	1.11	1.06–1.16	<0.001	1.10	1.01–1.18	<0.05
Shoe types						
Total	–	–	<0.01			
Sneakers	1.00 (reference)	1.00 (reference)	–			
Natural leather	0.68	0.34–1.36	0.275			
Synthetic leather	0.60	0.36–0.99	<0.05			
Sandals	0.68	0.23–2.00	0.486			
Boots	0.11	0.05–0.29	<0.001			
Slippers	0.52	0.08–3.22	0.482			
Cloth	1.17	0.19–7.25	0.867			
Mean monthly temperature in local area	1.09	1.06–1.12	<0.001			
Mean monthly humidity in local area	1.04	0.99–1.09	0.153			

Analyzed using logistic regression. CI, confidence interval; TP, tinea pedis.

Multivariate analysis indicated that the dew point inside the footwear was a significant risk factor for TP (Table 2). For each 1°C increase in dew point inside the footwear, the risk for TP increased by 1.1‐fold (OR, 1.10; 95% CI, 1.01–1.18; *P* < 0.05). For TU, temperature was a significant factor (*P* < 0.05; Table S2). The dew point was also a significant factor in fungal foot disease (OR, 1.09; 95% CI, 1.02–1.17; *P* < 0.05; Table S3).

The internal footwear environments of the TP subjects had significantly higher temperature, humidity, dew point and high‐temperature/high‐humidity environments. In cases with fungal foot disease, the ratio of subjects who wore high‐temperature/high‐humidity footwear was significantly higher. For both TP and fungal foot disease, the internal footwear environment was prone to becoming humid.

### Humidity of the interdigital area

The average humidity of the fourth interdigital area of the 10 male subjects during the summer was 80%, with average maximum humidity of 84%, maximum humidity of 91% and average dew point of 31°C.

### Optimal cut‐off point of TP

For the measurement conditions of the internal environments of the footwear, the optimum cut‐off point by the ROC analysis to discriminate between TP and non‐TP was humidity of 75.05% (OR, 3.03) and dew point of 24.56°C (OR, 3.62) for TP only, and humidity of 74.94% (OR, 3.19) and dew point of 23.14°C (OR, 3.19) for fungal foot disease.

## Discussion

Previous epidemiological and clinical studies have indicated a relationship between TP and footwear. However, it has not been scientifically proven that the internal environment of footwear and primary climatological factors have an effect on TP risk. To the best of my knowledge, this study is the first to measure the internal environment of footwear and the external environment, as well as assess how they relate to TP and TU incidences. In the present study, measurements were made of the internal footwear environment in the patients’ everyday life setting. These measurements were made during patient hospital visits and associations with TP and TU infections were determined. The results indicate that TP incidence is strongly affected by the internal environment of the footwear, that a significant correlation was found between footwear's internal environment and the season. Moreover, high‐temperature/high‐humidity footwear environments with a high dew point showed a significantly higher prevalence of TP.

The footwear's internal environment was more conducive for TP in men than in women.

The findings showed that the dew point is a useful indicator of a footwear's internal environment that easily becomes stuffy and is favorable for TP.

### Season and internal footwear environment

Although many epidemiological surveys and clinical experiences show a correlation of TP incidence with seasonal changes,^13^ no evidence‐based studies have been conducted to understand why TP becomes more prevalent in the summer and less so in the winter.

Typically, a region's climate has an influence on the footwear's internal temperature.^14^

The results presented herein demonstrate a significant correlation between high‐temperature/high‐humidity and high dew point of the footwear's internal environment and the season (i.e. higher in summer/fall than in winter/spring).

Interestingly, 42.3% of TP patients wore footwear that likely have a high‐temperature/high‐humidity internal environment during summer.

### Footwear type and internal footwear environment

Many studies have reported the relationship between the footwear's internal environment and footwear type.^15^, ^16^, ^17^ When comparing the internal environment between closed and non‐closed footwear, the closed‐type shoes showed significantly higher temperature and higher humidity inside the footwear. Thus, footwear type has important implications for TP patients, such as selecting the appropriate types and materials of the footwear. Furthermore, the temperature/humidity depended on the footwear material, with shoes with cloth material having the highest levels, followed by synthetic leather, artificial leather, natural leather, athletic and mesh. Thus, while shoes with cloth material are extremely comfortable, they have a stuffy internal environment; hence, these shoes have high TP risk. Furthermore, office workers tend to wear synthetic leather (32.7%) and natural leather (12.9%) shoes, which are both closed‐type shoes that are stuffy and have high TP risk.

The type of shoes (leather shoes, safety shoes, high heels) is related to TP;^7^ thus, if the footwear type is changed, then the internal humidity and temperature may dramatically decrease. Specifically, instead of wearing closed‐type shoes, such as natural and synthetic leather shoes, wearing open footwear such as sandals and slippers may decrease the risk of TP. This study did not identify the type of footwear that patients with TP were wearing because the research was not conducted in a controlled environment. Instead, the internal environment of footwear was measured randomly for patients that came into the clinic. It was observed that rather than the type of footwear, other “human” factors were associated with the development and progression of TP and TU. These include individual human factors such as underlying disease, physiological characteristics and body structure, as well as factors such as lifestyle and the way footwear is maintained and worn.

### Sex and internal footwear environment

Many studies have reported the relationship between sex and TP. These include studies reporting that TP occurs more frequently in men,^3^, ^4^, ^5^, ^9^, ^11^, ^18^, ^19^, ^20^, ^21^, ^22^ whereas other studies reported no significant difference.^23^, ^24^ The results of the present study demonstrated that a significant difference in the footwear's internal environment was found between men and women, with the internal humidity and dew point being significantly higher in the footwear worn by men. The frequency with which men wore footwear with a high‐temperature/high‐humidity internal environment was also higher. The fact that a high percentage of men wore their footwear continuously may also contribute to the increased TP incidence.^25^, ^26^, ^27^ The reason why TP occurs more frequently in men is still unclear, but human factors (e.g. men tend to sweat more than women),^28^ occupational factors and factors related to lifestyle habits may contribute to the difference between men and women in terms of frequency of footwear use and TP incidence.

### Conditions required for dermatophyte infection

Whether temperature or humidity is a critical requirement for dermatophyte infection is currently under debate. However, no studies have elucidated the role of the footwear's internal environment in *Trichophyton* penetration into the stratum corneum of a host. *In vitro* studies on invasion and proliferation of *Trichophyton rubrum* and *Trichophyton mentagrophytes*, which are major pathogens causing tinea in the stratum corneum, show that both fungi enter the stratum corneum within a day at two different humidity conditions (in an incubator at 35°C: 90% humidity and 100% humidity). The invasion rate of the dermatophytes was slower at 80% humidity,^29^ but increased proportionally with increasing humidity.^30^, ^31^ *T. rubrum* and *T. mentagrophytes* infiltration was absent at 85% humidity or less, and humidity needed to be at least 90% for dermatophytes to infiltrate and proliferate in the stratum corneum.^29^, ^32^ However, *T. mentagrophytes* were able to penetrate the stratum corneum in cases of skin injury even at 70% humidity.^30^

Further, my results show that the optimal cut‐off point for discriminating between the risk of tinea pedis and non‐tinea pedis is 75% humidity for both patients with TP only and fungal foot disease; humidity and dew point can be assumed to be even higher in the interdigital area and areas close to the skin surface of the foot. Indeed, the average humidity of the fourth interdigital area in the summer was 80%, average maximum humidity was 84%, maximum humidity was 91% and average dew point was 31°C. A humid environment is important for the development of tinea infections;^25^, ^26^, ^28^, ^30^, ^31^, ^33^ however, high humidity was not the major factor contributing to the development of tinea manuum, tinea corporis and tinea capitis,^31^ but rather the high‐temperature and high‐humidity environment. Based on these studies, the following three factors can be considered necessary for tinea development: an environment with high temperature and high humidity (temperature and humidity), sufficient dermatophyte adhesion time (time) and fine scratches on the skin or nails (injury).^4^, ^32^, ^33^, ^34^, ^35^, ^36^ Reducing these three conditions can prevent exacerbating TP infections. In addition, the length of time that footwear is worn is also thought to be a risk factor for TP.

This study has several limitations. First, the study was conducted in a single institution. Second, the research was performed with arbitrary footwear (footwear worn at the visit) and the effect of changing the footwear by the same patients on different occasions was not examined. Finally, the conditions including sex, age, shoe type and experimental environment in this study are also not uniform, as they would be in a controlled experimental study, which is important to completely understand the relationship between footwear type and TP and TU incidences. Thus, a multi‐institutional study with a large sample size should be conducted to verify the results from this study. Verification of the results in countries with climate and lifestyle habits different from those in Japan is also necessary.

In conclusion, the internal environment of footwear is a risk factor for TP and TU. The findings confirmed that the footwear's internal environment is affected by the season. Moreover, frequency of TP among men is related to the interior environment of footwear. The dew point is an appropriate index for evaluating temperature and humidity environments in relation to TP. Although this study does not contribute directly to clinical practise, it may enable dermatologists to advise patients on how to wear and maintain footwear, rather than limiting consultations to simply prescribing topical and internal agents. Consequently, dermatologists may play an important role in TP treatment and prevention.

## Conflict of Interest

None declared.

## Supporting information


**Methods S1.** Submission methods**.**
**Figure S1**. Internal temperature, humidity and dew point of the footwear during cold and warm seasons.
**Table S1.** Demographic and clinical characteristics.
**Table S2.** Tinea unguium (TU) risk factor analysis.
**Table S3.** Fungal foot disease risk factor analysis.
