# Supplementary material for: Internal environment of footwear is a risk factor for tinea pedis
Source: J Dermatol. 2019 Aug 22;46(11):940–6. doi: 10.1111/1346-8138.15060 (PMC6900014; doi:10.1111/1346-8138.15060)
Supplement: Supplementary file 1 — Methods S1. Submission methods. Figure S1. Internal temperature, humidity and dew point of the footwear during cold and warm seasons. Table S1. Demographic and clinical characteristics. Table S2. Tinea unguium (TU) risk factor analysis. Table S3. Fungal foot disease risk factor analysis. [file JDE-46-940-s001.docx]

**Supplementary Material**

**Internal Environment of Footwear is a Risk Factor for Tinea Pedis**

Yukio Sasagawa, MD

Sasagawa Dermatological Clinic, Jyoto-ku, Osaka, Japan

Corresponding author: Yukio Sasagawa

Sasagawa Dermatological Clinic,

1-1-15, Shinkita, Jyoto-ku, Osaka 536-0015, Japan

Phone: 81 06 6931 8009

Fax: 81 06 6914 2656

E-mail: [ysasa@mtf.biglobe.ne.jp](mailto:ysasa@mtf.biglobe.ne.jp)

**Content**

1. Supplementary Methods: Submission methods
2. Supplementary Table 1. Demographic and clinical characteristics
3. Supplementary Table 2. TU risk factor analysis
4. Supplementary Table 3. Fungal foot disease risk factor analysis
5. Supplementary Figure 1. Internal temperature, humidity, and dew point of footwear in the cold and warm seasons.
6. Supplementary Figure 2. Mean humidity and temperature in different types of footwear.

**Supplementary methods**

**Submission methods**

**Statistical analysis**

The results of the continuous variables are presented as mean ± standard deviation. The analysis was performed using either the one-way analysis of variance (ANOVA) or the t-test. Using the Shapiro-Wilk test and by observing the actual data distribution on a histogram, it was determined that the data distribution was normalized.

The relationship between the internal temperature of the footwear and the monthly mean temperature in the local area (the city of Osaka), and the relationship between the internal humidity of footwear and the monthly mean humidity in the local area were subjected to a single correlation analysis using the parametric Pearson’s correlation coefficient. The significance of the correlation coefficient was evaluated by testing the correlation coefficient.

The Tukey’s honestly significant difference (HSD) method and Dunnett’s T3 test were used to perform parametric multiple comparisons of the temperature, humidity, and dew point inside the footwear during different seasons. Comparisons were performed for all inter-group combinations. These tests were also used to compare the temperature, humidity, and dew point inside the footwear according to the type of footwear. Comparisons were performed for all inter-group combinations.

Comparisons of the high-temperature/high-humidity environment inside the footwear by season (except for high-temperature/high-humidity vs. high-temperature/high-humidity) were performed using a contingency table test (chi-squared test of independence) that compared the independent nominal scale data (frequency data). This test was also used to compare the high-temperature/high-humidity environment inside the footwear by footwear type (except for high-temperature/high-humidity vs. high-temperature/high-humidity) and to compare the high-temperature/high-humidity environment inside the footwear by sex. Similarly, this test was used to compare the high-temperature/high-humidity environments inside the footwear between the TP and non-tinea groups, between the TU and non-tinea groups, between the both and non-tinea groups, and between the fungal foot disease and non-tinea groups (except for high-temperature/high-humidity vs. high-temperature/high-humidity).

Comparisons of the temperature, humidity, and dew point inside footwear by sex were performed using either the t-test (used to compare parametric independent data between two groups) or Welch’s t-test.

Patients’ background characteristics were analyzed as follows: the one-way ANOVA or the t-test was used to analyze age, mean temperature inside the footwear (°C), mean humidity inside the footwear (% RH), mean monthly temperature in the city of Osaka, mean monthly humidity in the city of Osaka, and mean estimated dew point (°C). The contingency table test (chi-squared test of independence) was used to analyze sex, footwear type, and high-temperature/high-humidity environment inside the footwear (except for high-temperature/high-humidity vs. high-temperature/high-humidity).

Analysis of the risk factors of TP was performed by forced selection of variables using the forced entry method in the selection of variables for univariate analysis. The significance of the factors was determined using the Wald test. Variable selection for multivariate analysis was performed using the ‘variable increase (forward) stepwise method (likelihood ratio) – (probability of inclusion ≤.050, probability of exclusion ≤.100)’. The significance of the factors was determined using logistic regression. Significance in all tests was uniformly set at 5%.

The results of the categorical variables are shown as a percent (%), and their range was analyzed using the Fisher’s exact test. The data conformed to the Gauss distribution. Parametric multi-comparisons were performed using the Tukey HDS method and Dunnett’s T3 test, and univariate analysis of the relation between the internal environment of footwear and the season was performed using Pearson’s correlation coefficient. Factors (e.g., season, sex, age, internal environment of footwear, local climate) shown to be significant in Wald’s test as a result of univariate analysis using logistic regression were then included in the multivariate analysis to determine their significance. The standard of significance was set to 5%.

**Online-only tables**

**Supplementary Table 1.** Demographic and clinical characteristics

|  | **Total**  **(n = 420）** | **Non-tinea**  **(n = 213)** | **TP†**  **(n = 165)** | **TU†**  **(n = 92)** | **Fungal foot disease**  **(n = 207)** |
| --- | --- | --- | --- | --- | --- |
| **Sex** |  |  |  |  |  |
| **Men** | 190 (45.2%) | 84 (39.4%) | 89 (53.9%) | 46 (50.0%) | 106 (51.2%) |
| **Women** | 230 (54.8%) | 129 (60.6%) | 76 (46.1%) | 46 (50.0%) | 101 (48.8%) |
| **Age (years)** |  |  |  |  |  |
| **<30** | 90 (21.4%) | 65 (30.5%) | 20 (12.1%) | 9 (9.8%) | 25 (12.1%) |
| **≥30 and <60** | 187 (44.5%) | 106 (49.8%) | 71 (43.0%) | 28 (30.4%) | 81 (39.1%) |
| **≥60** | 143 (34.0%) | 42 (19.7%) | 74 (44.8%) | 55 (59.8%) | 101 (48.8%) |

TP†: TP only (115) and Both (TP only and TU only; 50)

TU†: TU only (42) and Both (TP only and TU only; 50)

Fungal foot disease: TP only or TU only or both (207)

Abbreviations: TP, tinea pedis; TU, tinea unguium

**Supplementary Table 2.** TU risk factor analysis

|  | | **Univariate analysis** | | | **Multivariate analysis** | | |
| --- | --- | --- | --- | --- | --- | --- | --- |
| **Variable** | | **Odds ratio** | **95% CI** | **P value** | **Odds ratio** | **95% CI** | **P value** |
| **Sex (male)** | | 1.54 | 0.94 - 2.51 | 0.088 |  |  |  |
| **Age groups** | Total | - | - | - | - | - | <0.05 |
|  | <30 years | 1.00 (reference) | 1.00 (reference) | - | 1.00 (reference) | 1.00 (reference) | - |
|  | ≥30 and <60 years | 1.91 | 0.85 - 4.30 | 2.48 | 2.48 | 0.44 - 14.14 | 0.305 |
|  | ≥60 years | 9.46 | 4.23 - 21.14 | 6.36 | 6.36 | 1.17 - 34.59 | <0.05 |
| **Internal high temperature/high humidity** | | 1.36 | 0.75 - 2.45 | 0.308 |  |  |  |
| **Internal dew point** | | 1.07 | 1.01 - 1.13 | <0.05 |  |  |  |
| **Footwear types** | Total | - | - | <0.01 |  |  |  |
|  | Sneakers | 1.00 (reference) | 1.00 (reference) | - |  |  |  |
|  | Natural leather | 0.66 | 0.29 - 1.50 | 0.319 |  |  |  |
|  | Synthetic leather | 0.46 | 0.24 - 0.88 | <0.05 |  |  |  |
|  | Sandals | 0.90 | 0.27 - 2.95 | 0.856 |  |  |  |
|  | Boots | 0.07 | 0.02 - 0.31 | <0.001 |  |  |  |
|  | Slippers | 0.95 | 0.15 - 6.00 | 0.961 |  |  |  |
|  | Cloth | 3.58 | 0.66 - 19.46 | 0.140 |  |  |  |
| **Mean monthly temperature in the local area** | | 1.10 | 1.05 - 1.14 | <0.001 | 1.06 | 1.00 - 1.13 | <0.05 |
| **Mean monthly humidity in local area** | | 1.11 | 1.04 - 1.18 | <0.01 |  |  |  |

CI: confidence interval

Analyzed using logistic regression analysis.

**Supplementary Table 3.** Fungal foot disease risk factor analysis

|  | | **Univariate analysis** | | | **Multivariate analysis** | | |
| --- | --- | --- | --- | --- | --- | --- | --- |
| **Variable** | | **Odds ratio** | **95% CI** | **P value** | **Odds ratio** | **95% CI** | **P value** |
| **Sex (men)** | | 1.61 | 1.09–2.37 | <0.05 |  |  |  |
| **Age groups** | Total | – | – | <0.001 | - | - | <0.01 |
|  | <30 years | 1.00 (reference) | 1.00 (reference) | – | 1.00 (reference) | 1.00 (reference) | - |
|  | ≥30 and <60 years | 1.99 | 1.15 - 3.42 | <0.05 | 3.93 | 0.98 - 15.75 | 0.054 |
|  | ≥60 years | 6.25 | 3.48 - 11.22 | <0.001 | 10.19 | 2.60 - 39.94 | <0.001 |
| **Internal temperature** | | 1.15 | 1.08-1.23 | <0.001 |  |  |  |
| **Internal humidity** | | 1.03 | 1.01-1.05 | <0.01 |  |  |  |
| **Internal high-temperature/high-humidity** | | 1.89 | 1.20 - 2.98 | <0.01 |  |  |  |
| **Internal dew point** | | 1.10 | 1.05 - 1.14 | <0.001 | 1.09 | 1.02 - 1.17 | <0.05 |
| **Footwear types** | Total | – | – | <0.01 |  |  |  |
|  | Sneakers | 1.00 (reference) | 1.00 (reference) | – |  |  |  |
|  | Natural leather | 0.76 | 0.40 - 1.47 | 0.419 |  |  |  |
|  | Synthetic leather | 0.61 | 0.37 - 1.00 | <0.05 |  |  |  |
|  | Sandals | 0.85 | 0.31 - 2.29 | 0.747 |  |  |  |
|  | Boots | 0.13 | 0.06 - 0.31 | <0.001 |  |  |  |
|  | Slippers | 0.68 | 0.13 - 3.50 | 0.644 |  |  |  |
|  | Cloth | 2.72 | 0.56 - 13.31 | 0.217 |  |  |  |
| **Mean monthly temperature in the local area** | | 1.09 | 1.06–1.12 | <0.001 |  |  |  |
| **Mean monthly humidity in the local area** | | 1.04 | 0.99–1.09 | <0.01 |  |  |  |

CI: confidence interval.

Analyzed using logistic regression analysis.

**Online-only figures**


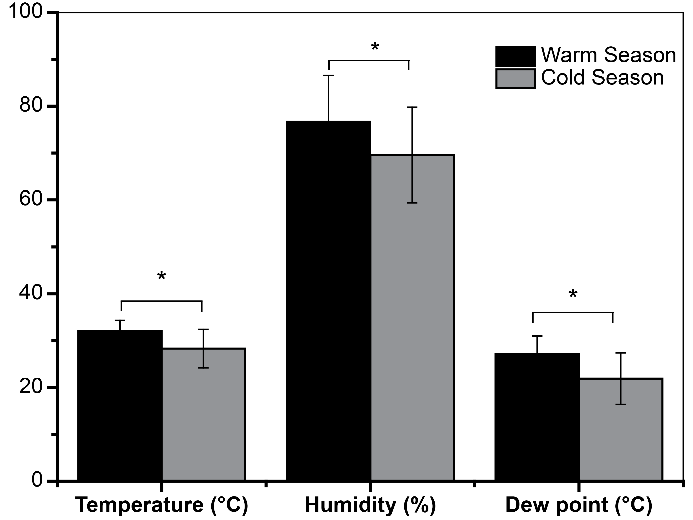


**Supplementary Figure 1. Internal temperature, humidity, and dew point of the footwear during cold and warm seasons.**

The mean temperature inside the footwear was significantly higher in fall and summer than in spring and winter (warm season: 32.0 °C ± 2.3 °C, cold season: 28.3 °C ± 4.1 °C, P < 0.001). The humidity inside the footwear was also significantly higher in the warm season than in the cold season (warm season: 76.7% ± 9.8%, cold season: 69.6# ± 10.2%, P < 0.001). A similar trend was found for dew point (warm season: 27.2 °C ± 3.8 °C, cold season: 21.9 °C ± 5.5 °C, P < 0.001.


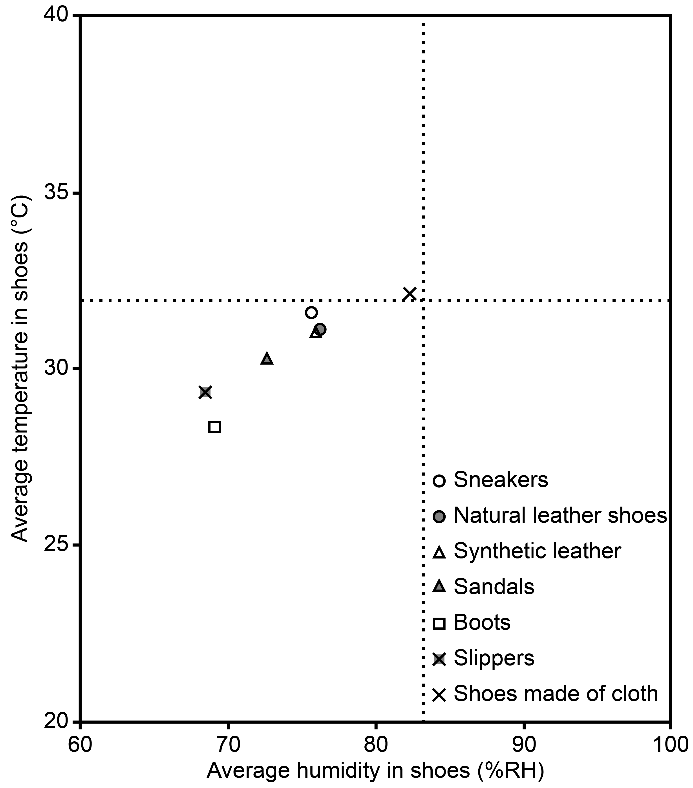


**Supplementary Figure 2. Mean humidity and temperature in different types of footwear.**

Cloth footwear had the highest temperature, humidity, and dew point values and was prone to mustiness. Closed footwear (e.g., natural leather footwear, synthetic leather footwear, sneakers) had higher temperature, humidity, and dew point values than open footwear (e.g., sandals, slippers). Boots were worn during the winter and therefore had low temperature, humidity, and dew point values.
